# Production of pyrolytic oil from ULDP plastics using silica-alumina catalyst and used as fuel for DI diesel engine

**DOI:** 10.1039/d0ra07073d

**Published:** 2020-10-12

**Authors:** Soundararajan Gopinath, P. K. Devan, K. Pitchandi

**Affiliations:** Department of Mechanical Engineering, RMK College of Engineering and Technology Puduvoyal – 601 206 Tamil Nadu India gp.nath4@gmail.com; Department of Mechanical Engineering, Sri Venkateswara College of Engineering Sriperumbudur – 602 117 Tamil Nadu India

## Abstract

A rapid increase in the use of non-biodegradable plastics and their disposal after use has had a detrimental impact on the environment. Used plastics (used low-density polyethylene – ULDP) were selected as feedstock for the extraction of pyrolytic oil. The pyrolysis process was carried out in a semi-batch reactor with a silica alumina catalyst in the existence of fluidizing gas N_2_ in a reactor at 500 °C for 60 min. The maximum liquid, gas, and char yields were 93.5 wt%, 5.4 wt%, and 1.1 wt%, respectively. Experimental analysis was carried out to obtain their functional and structural groups by FT-IR and the carbon distribution was identified by GC-MS analysis. The blends of 20%, 40%, 60%, 80%, and 100% on a volume basis were chosen for the detailed study. For the pyrolytic blends, the combustion, performance, and emission characteristics were tested at different engine loads. During combustion, the heat release rate was extremely high for neat ULDP oil because of the high energy content and a higher cetane index. The efficiency of ULDP20 was higher than in other blends, whereas NOx and smoke emissions of ULDP20 were lower among the blends but higher than diesel. ULDP20 performed similarly as diesel. Hence, ULDP20 is recommended as a fuel for the diesel engine.

## Abstract 2

Lower acidity ratio of SA catalyst influences higher yield of ULDP oil and is suggested as a novel fuel for unmodified diesel engine.

## Graphical abstract

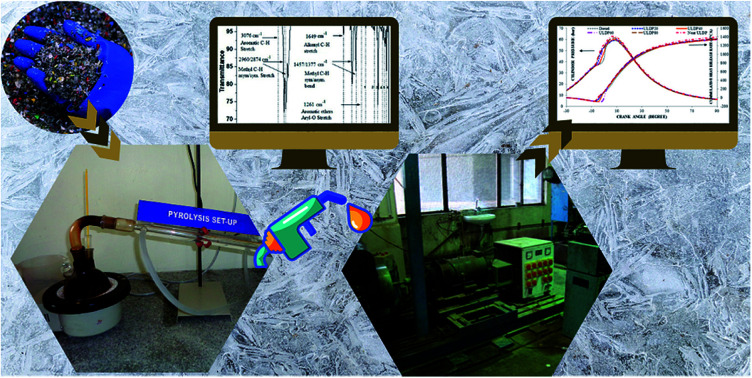

## 1. Introduction

The tremendous use of different types of non-biodegradable plastics like low-density polyethylene (LDPE), high-density polyethylene (HDPE), polystyrene (PS), polypropylene (PP) and polyvinyl chloride (PVC) in various applications results in 5.56 million tons of plastic waste every year. In addition, fossil fuel demand and prices are increasing. To balance the demand, research is focused on developing alternative fuels.^1,2^ Experimental investigation has been performed on HDPE oil derived from waste plastic grocery bags. The chemical structure of HDPE pyrolysis oil contains 94.0% aliphatic, paraffinic hydrogens, and a smaller amount of olefinic hydrogen. Based on the property analysis, it is observed that all the chemical properties except lubricity are similar to those of diesel. ^3^ Experimental analysis was carried out on plastic oil blends (25%, 50%, 75%) in a DI diesel engine without any modification. It was found that the brake thermal efficiency was improved in lower plastic oil blends compared to 100% neat plastic oil and higher blends due to low viscosity and density. Emissions such as smoke and nitrogen oxides (NOx) were considerably lower by 22% and 17% respectively for P25 ^4^.

The combustion and emission characteristics of pyrolysis oil with the addition of 5% and 10% diethyl ether (DEE) in a water-cooled DI diesel engine were examined. The brake thermal efficiency (BTE) was improved compared to that of plastic oil, whereas the peak pressure was reduced by the addition of DEE.^5^ Due to the addition of DEE to plastic oil, the cetane number was increased, thereby, a reduction in NOx and carbon monoxide (CO) emissions along with an increase in hydrocarbon emission (HC) were observed. The addition of DEE improved the oxygen content in the blends of plastic pyrolytic oil, resulting in excellent combustion. An engine fueled with plastic oil with an additive of di-ethyl ether demonstrated a significant rise in BTE, exhaust gas temperature (EGT), and reduced CO emissions.^6,7^

An experiment was conducted on the CI engine with waste plastic oil to ensure its engine characteristics. It was observed that while using 100% waste plastic oil in the diesel engine, emissions from NOx, CO, hydrocarbons, and smoke increased by 25%, 5%, 15% and 40%, respectively, compared to diesel fuel.^8^ However, a higher brake thermal efficiency was observed when compared to diesel fuel.

Researchers investigated the combustion and output characteristics of rice bran methyl ester (RBME) and plastic oil (PO) in a CI engine compared with regular diesel fuel.^9^ They reported that with 100% neat plastic oil, the engine was able to run, but the performance and combustion characteristics were inferior due to its longer ignition delay and prolonged combustion duration.^10^ The NOx emissions were increased with neat (100%) plastic oil due to its higher heat release rate compared with diesel. The selective catalytic reduction technique was adopted to reduce the nitrogen oxides using a rhodium catalyst, resulting in an optimum temperature range of 240 °C to 280 °C and a reduction of NOx up to 30%.^11^ Higher blends of recycled waste plastic oil exhibit higher emissions from NOx and smoke. Supporting experiments were performed with the addition of pentanol to waste plastic oil with a retarded injection timing of 21 °CA BTDC and the adoption of 10% exhaust gas recirculation (EGR) reduced the smoke emission by 74.2% with a significant increase in NOx emission. The brake specific fuel consumption (BSFC) also improved by 3.2% at higher loads.^12,13^

The pyrolysis process was conducted with waste tyres at a maximum temperature of 500 °C in the presence of nitrogen gas (carrier gas) in the pyrolysis reactor to obtain the waste tyre pyrolysis oil. Experimental investigations were carried out at various loads with a 10% blend of waste tyre oil.^14^ The combustion duration was increased by up to 1.16% with a reduction in CO emissions and the brake thermal efficiency increased by 3.2% for a 10% blend.^15^ The plastic pyrolysis oils from mixed plastic wastes were obtained by the thermal depolymerization method.^16^ The performance, combustion, and emission characteristics were much closer to diesel fuel at lower (below 25%) loads. However, a higher load increase in the NOx and CO emission was abnormal due to the presence of the unsaturated double bonds in plastic oils.

The injection timing was increased up to 25 °CA bTDC, which reduced the smoke and NOx by 38% and 46%.^17^. Continuous tests on the DI engine fueled with neat (100%) plastic oil were conducted to evaluate the performance, combustion, and emission.^18^ The experimental results revealed that the engine performance was reduced due to wear on the piston. To improve the efficiency, the addition of standard diesel was required for a longer run of the diesel engine.

Many researchers have reported the conversion of used plastics into oil by the pyrolysis method and then used the oil as a fuel. However, the type of plastic being used was not discussed. In the current investigation, used low-density polyethylene (ULDP) was selected as a feedstock for oil extraction because of its abundant availability. Silica alumina (SA) was used as a catalyst for the production of pyrolytic ULDP oil in a semi-batch reactor. Then, ULDP oil was mixed with diesel in different proportions and also neat. Researchers have reported that a lower acidity ratio of the silica alumina catalyst leads to a higher liquid yield in the pyrolysis process. The physiochemical properties and fuel injection characteristics of blends, and neat ULDP oil were measured and compared with diesel. The types of functional groups were identified by Fourier transform infrared spectroscopy (FTIR), and gas chromatography-mass spectrometry (GC-MS) was conducted to investigate the presence of saturated and unsaturated compounds. To examine the suitability of the blended ULDP fuels, combustion tests were carried out on the DI diesel engine at different operating points. Further, the thermodynamic performance and exhaust emissions of the ULDP oil and its blends were found and compared with diesel. Since minimal research work has been carried out with 100% plastic oil, in the present investigation, an attempt was made and the results are compared between blends and diesel with neat ULDP oil. The main objective is to characterize and convert waste plastic to pyrolysis oil, which can provide an environmentally friendly diesel fuel.

## 2. Materials

### 2.1 Selection of feedstock

**Fig. 1:**
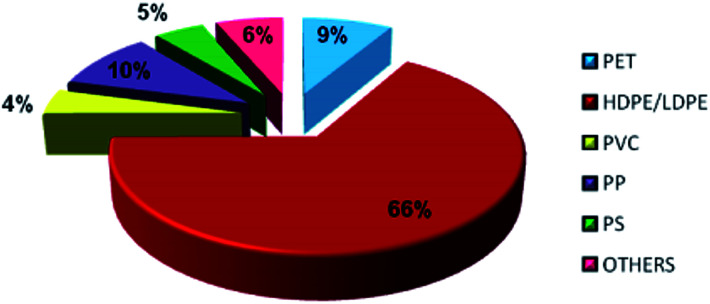
Composition of waste plastics in India.

In general, plastics are classified based on their polymers namely polyethylene terephthalate (PET), high-density polyethylene (HDPE), polyvinyl chloride (PVC), low-density polyethylene (LDPE), polypropylene (PP) and polystyrene (PS). The recent survey report of the Ministry of Human Resources and Development of India reveals that 94% of plastic wastes are thermoplastics and the remaining 6% of plastic wastes are thermoset plastics. The detailed analysis of plastic waste in India is shown in Fig. 1 and the percentages are described here: polyethylene terephthalate (PET) is 8.66% (5.69 kg perMetric Ton (MT)), high density polyethylene and low density polyethylene (HDPE and LDPE) is 66.91% (43.94 kg MT^−1^), polyvinyl chloride (PVC) is 4.14% (2.72 kg MT^−1^), polypropylene (PP) is 9.90% (6.50 kg MT^−1^), polystyrene (PS) is 4.77% (3.13 kg MT^−1^), and others are 6.43% (4.22 kg MT^−1^).^19^ This clearly shows that LDPE and HDPE are a major proportion of the waste plastic composition of about 66%.

**Fig. 2:**
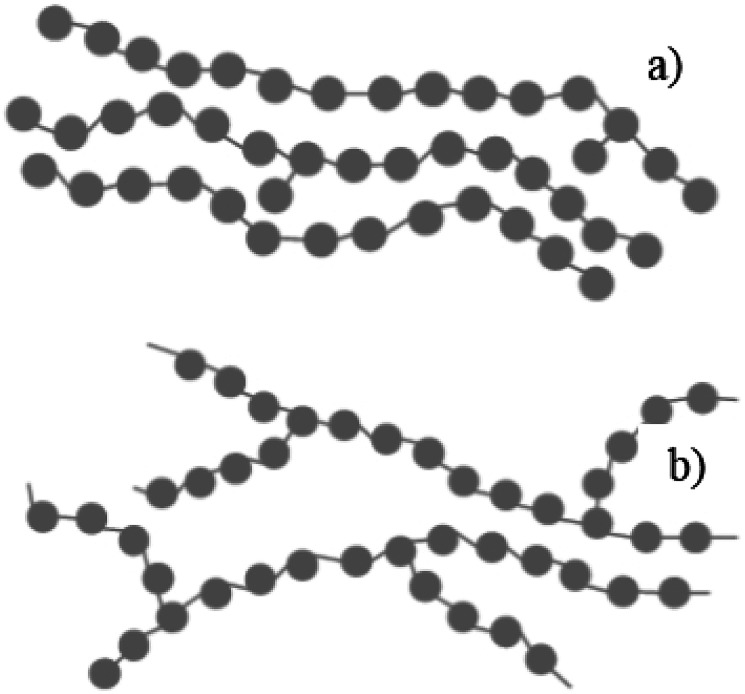
(a) HDPE structure (linear) (b) LDPE structure (branched).

LDPE has a branched structure, less dense and is flexible. In contrast, high-density polyethylene has a linear structure with high density chains, as shown in Fig. 2. LDPE has a very high impact strength compared to high-density polyethylene. Hence, the breaking of the HDPE chain is difficult when compared to LDPE. The catalytic cracking of low-density polyethylene chain is more feasible than HDPE because LDPE possesses a branched structure with lower density molecules in the chain. The pyrolysis process is carried out with low-density polyethylene as a feedstock and the maximum liquid yield is 85 wt% in the presence of a ZSM-5 catalyst at 500 °C with the reaction time of 60 min.^20^ Whereas, the HDPE feedstock provides a liquid yield of 80 wt% with the same catalyst and operating parameters.^21^ Considering the maximum pyrolytic liquid yield and its characteristics, LDPE was selected as a feedstock in this research work.

### 2.2 Selection of catalyst

Zeolite-based heterogeneous catalysts HZSM-5, HUSY, HMOR, and Hβ are preferred for the pyrolysis process because of their useful ion exchange characteristics. A non-zeolite heterogeneous catalyst, such as silica alumina and silicalite which have more electron-accepting aspects, is also used for the pyrolysis process. A fluid catalytic cracking (FCC) catalyst is composed of a non-zeolite and zeolite, such as a binder with silica-alumina (SA).^22^ This FCC catalyst is widely used in petroleum refinery to break the heavier chain in crude oils. The FCC silica alumina catalyst increases the rate of breaking chains to produce a high liquid and gas yield. A higher strength of acidity is determined by a higher ratio like 4.99, referred to as SA-1, whereas 0.27 is referred to as SA-2. A lower acidity silica alumina catalyst produces a higher liquid yield of 74.3 wt% for HDPE whereas for the SA-1 catalyst, the liquid yield is 67.8 wt% and for the ZSM-5 catalyst, the liquid yield is 49.8 wt%.^23^ Hence, in this research, FCC catalyst silica alumina is selected for the pyrolysis process.

### 2.3 Selection of fluidizing gas

Mostly inert gases such as hydrogen, helium, nitrogen, ethylene, propylene, and argon are used as fluidizing gases. The differences in molecular weight and molecular size of the inert gases impact the reactivity in the pyrolysis. The carrier gas, which has a lower molecular weight produces a higher liquid yield. With the feedstock of polypropylene, the yield (wt%) using an FCC catalyst at a reaction temperature of 450 °C was analyzed while varying the carrier gases. The molecular weights of hydrogen, helium, nitrogen, ethylene, propylene and argon are 2, 4, 28, 28, 42 and 37, which correspond to the liquid yields of 96.7%, 94.7%, 92.3%, 93.8%, 87.8% and 84.8%, respectively.^24^ H_2_ gas leads to a higher liquid yield compared to other gases, but in practice, the storage of hydrogen is a tedious process and also not cost-effective. When N_2_ is used as a carrier gas, only a 0.9% liquid yield difference between the H_2_ and N_2_ gases is observed. Hence, nitrogen was selected as the fluidizing gas in this research work.

## 3. Production and properties

### 3.1 Pyrolysis process

**Fig. 3:**
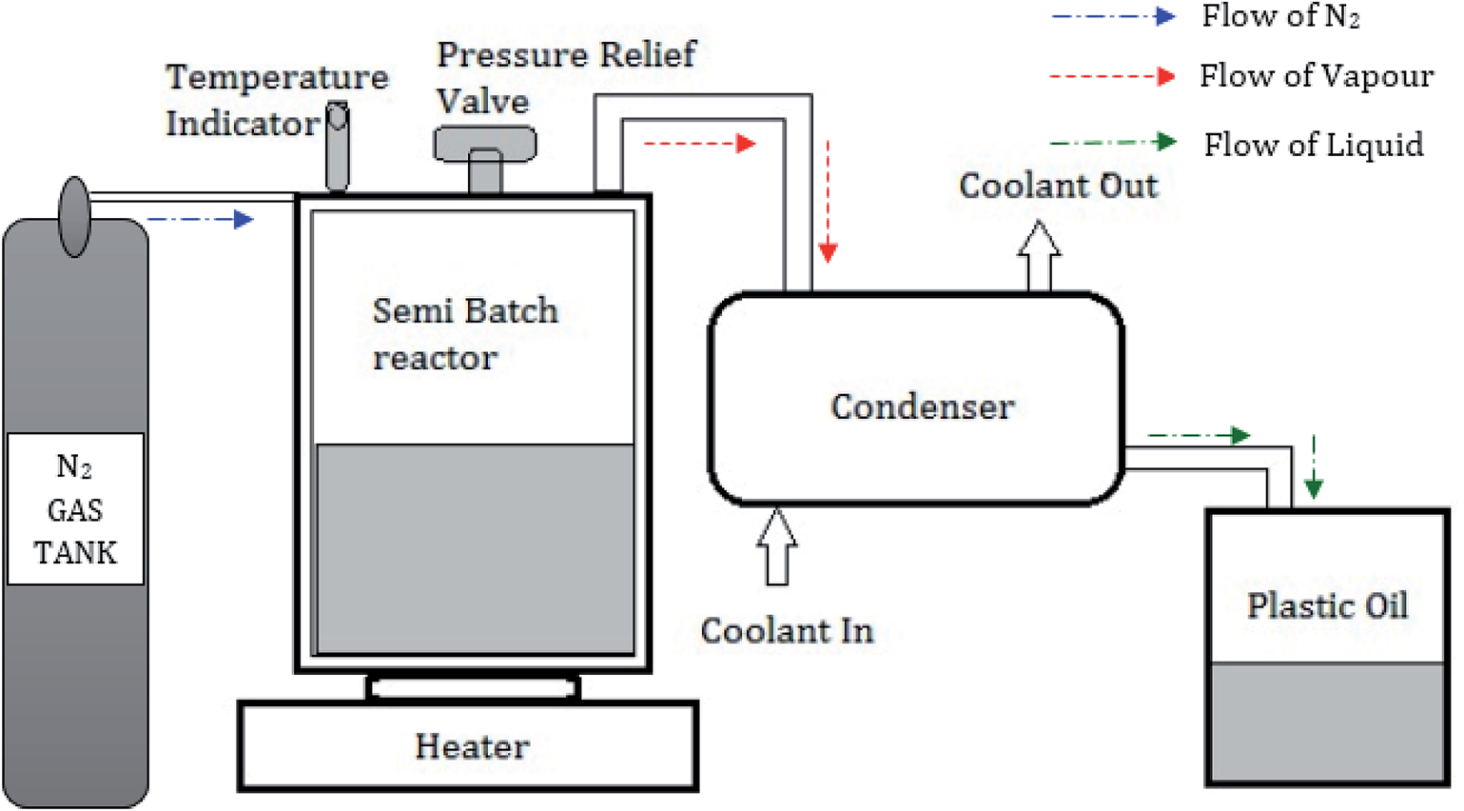
Schematic representation of Pyrolysis setup.

Used low-density polyethylene was chosen as the feedstock for oil extraction by the pyrolysis process among the various forms of plastic polymers. A semi-batch pyrolysis reactor with a 1500 mm diameter and 1200 mm height with a pressure relief valve was used in this investigation. A condenser with 1200 mm height and 1000 mm diameter consisting of 8 mm copper tubes was placed vertically through the series connection from the reactor.^25^ Water was used as a coolant through the outer shells of the condenser with the aid of a pump. An electric furnace was used to maintain the reactor at a constant temperature.^26^Fig. 3 shows the schematic representation of the pyrolysis setup. Used plastics collected from the dumped yards were dried in sunlight, cut into small pieces (10–20 mm^2^), and used to fill the reactor. Nitrogen gas was filled in the semi-batch reactor to ensure there was no oxygen inside the reactor. The reactor was placed on the electrical furnace, which is capable of varying temperatures from 100 °C to 600 °C. The silica alumina catalyst was added as 5 wt%, and the optimum temperature of 500 °C was maintained for 60 min. The gas, which comes out from the reactor, is sent to the condenser where gases are cooled, and liquid oil is collected in the tank. During this process, the liquid yield of 93.5 wt%, the gas yield of 5.4 wt%, and a char yield of 1.1 wt% were obtained. From 1 kg of used LDPE plastics feedstock, 0.9 liters of pyrolytic oil was extracted.

### 3.2 Physio-chemical properties

#### 3.2.1 Physical properties

The physical properties of diesel, neat ULDP oil (100% of ULDP oil), and its blends were obtained as per ASTM standards, and the results are shown in Table 1. The calorific value of neat (100%) ULDP oil is 42.9 MJ kg^−1^, which is higher than diesel fuel, tested as per ASTM D240 (Make: Toshniwal; Model: CC-01/M3). The density of neat ULDP oil is 784 kg m^−3^ as obtained at ASTM D4052 (Make: Anton Paar; Model-4500M), which is lesser than standard diesel at the temperature of 15 °C. The flashpoint was obtained through a Pensky Marten Flash Point Apparatus (Make: Tanaka; Model-apm-8) as per ASTM D93. A lower flashpoint was observed when compared to diesel fuel. Many researchers stated that plastic pyrolytic oil has a low flash and fire point. Kinematic viscosity was determined using a viscometer bath (Make: Tamson) as per ASTM D 445, showing a lower kinematic viscosity for neat ULDP oil when compared to standard diesel fuel. The calculated Cetane Index was obtained as per ASTM D976 and it describes the 2-point method. In this method, the density and the mid-boiling temperature were used to calculate the Cetane Index by using the equation below.^27^ The calculated Cetane Index is 49 for neat ULDP oil is higher than that of diesel. Many researchers have calculated the Cetane Index number by using the 2-point method and reported as per ASTM D976.^28^CCI = 454.74 − 1641.416*D* + 774.74*D*^2^ − 0.554*B* + 97.803 log^2^where, CCI – calculated cetane index, *D* – density at 15 °C g ml^−1^, *B* – mid boiling temperature °C.

**Table 1:** Physical properties of oil sample

Physical properties	Method	Diesel	Neat ULDP oil	ULDP20	ULDP40	ULDP60	ULDP80
Calorific value (mJ kg^−1^)	ASTM D 240	42.5	42.9	42.58	42.66	42.74	42.82
Density @ 15 °C (kg m^−3^)	ASTM D4052	807	784	802.4	797.8	793.2	788.6
Ash content (wt%)	ASTM D482	0.01	0.02	0.012	0.014	0.016	0.018
Flashpoint (°C)	ASTM D93	52	6	42.8	33.6	24.4	15.2
Kinematic viscosity (mm^2^ s^−1^) @ 40 °C	ASTM D 445	2.14	0.82	1.876	1.612	1.348	1.084
CCI (calculated cetane index)	ASTM D976	46	49	46.6	47.2	47.8	48.4

#### 3.2.2 FTIR analysis of neat ULDP oil

**Fig. 4:**
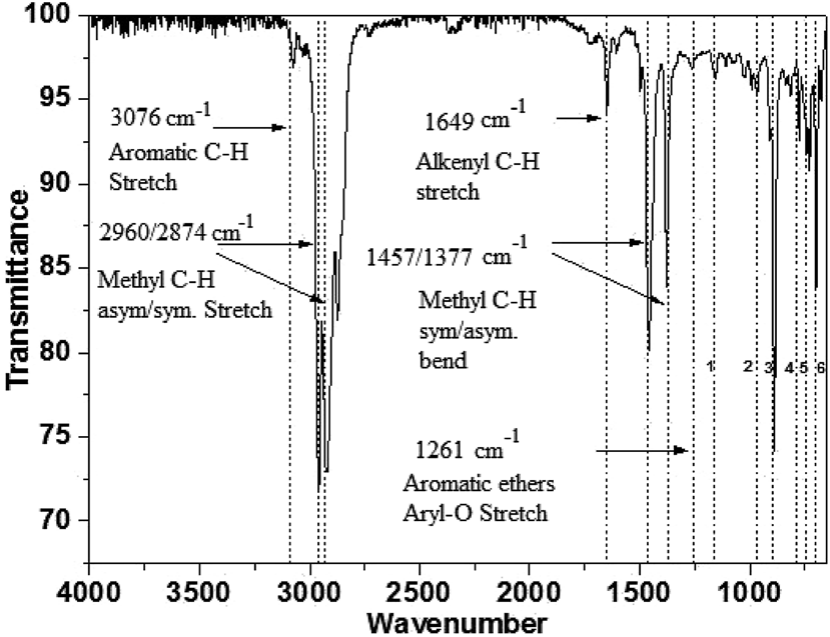
FT-IR spectra of neat ULDP oil.

The functional groups present in the neat ULDP oil were analyzed by using Fourier transform infrared spectroscopy (FT-IR). FT-IR (Make-Agilent, Model-Cary 630) as per the ASTM E1252 FT-IR test was used for neat ULDP oil, and the variations of spectral regions were recorded from 400 to 4000 cm^−1^ at the resolution of 8 cm^−1^. The test was carried out in two stages; in the first stage, the analysis of functional groups is inefficient, and thereby at the second stage, the absorption of the column provides better results than the early stage.^29^ The presence of aromatic C–H compounds in the neat ULDP oil was identified using peak analysis in Fig. 4 at 775 cm^−1^, and 3076 cm^−1^ whereas alkyl-substituted ether C–O are present at 1155 cm^−1^. Some of the variations of peaks beyond Fig. 4 are listed in Table 2. The absorption peaks at 1261 cm^−1^ and 1649 cm^−1^ correspond to aromatic ethers as aryl-O stretching and alkenyl C–H stretching. The absorption peaks that vary from 1377 cm^−1^ to 1457 cm^−1^ and 2960 cm^−1^ to 2874 cm^−1^ are attributed to the methyl C–H symmetric and asymmetric bending.^30^ The existence of the functional groups and compounds is confirmed using GC-MS analysis, as described in section 3.2.3, and it showed that the saturated and unsaturated compounds in neat ULDP oil are similar to standard diesel.

**Table 2:** FT-IR peak analysis of neat ULDP oil not shown in Fig. 2

Wavenumber	Functional group
1155 cm^−1^	Alkyl-substituted ether, C–O stretch
965 cm^−1^	*Trans* C–H out-of-plane bend
887 cm^−1^	Vinylidene C–H out-of-plane bend
775 cm^−1^	Aromatic C–H out-of-plane bend
728 & 697 cm^−1^	*Cis* C–H out-of-plane bend

#### 3.2.3 GC-MS analysis of neat ULDP oil

**Fig. 5:**
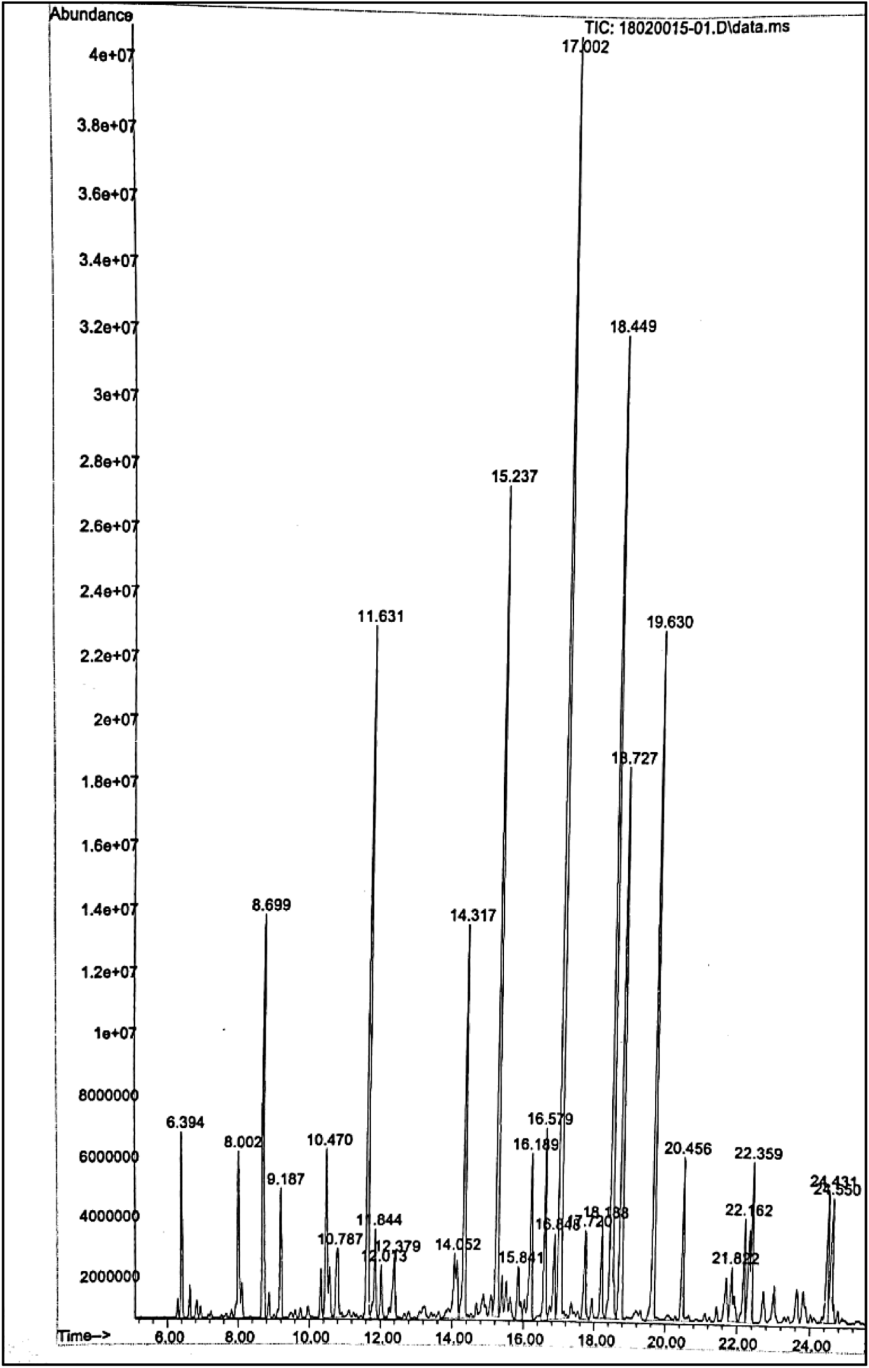
GC-MS plot of neat ULDP oil.

Neat ULDP oil was investigated by GC-MS to identify the molecular formula and compounds. The carbon distribution mass percentage and detailed compound analysis of neat ULDP oil is shown in Tables 3 and 4. The peak distributions of the GC-MS study is shown in Fig. 5. The results revealed that a higher number of alkanes and alkenes were found in neat (100%) ULDP oil. From GC-MS analysis, it was found that neat ULDP oil is composed of 20.74% C_6_–C_9_, 70.27% C_10_–C_15_, 22.68% C_16_–C_19_ and 7.03% > C_20_ carbon compounds. It clearly shows that heavier carbon compounds are very low and also higher concentrations of aromatic compounds are found.^31^ It also consists of 4.53% oxygenated compounds such as furan, acetyl cyclopentane, and 2-ethyl-2-methyl-2-hepten-4-one that are found in the analysis. The compounds present in neat ULDP oil are similar to diesel fuel. Hence, neat ULDP oil is suitable for the diesel engine in the direct form or blended form with diesel.

**Table 3:** Carbon distribution for neat ULDP oil through GC-MS analysis

Composition	Percentage (%)
C_6_–C_9_	20.74
C_10_–C_15_	64.69
C_16_–C_19_	12.18
>C_20_	2.37

**Table 4:** GC-MS Compound analysis of LDPE oil sample by catalytic pyrolysis

Number of peaks	Retention time, min	Area, %	Molecular formula	Compound name	Molecular structure
1	6.394	1.51	C_6_H_6_	Benzene	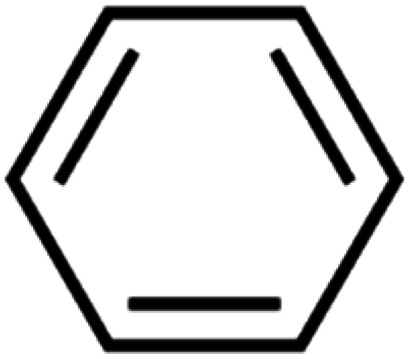
2	8.002	2.13	C_6_H_8_O	Furan, 2-ethyl-	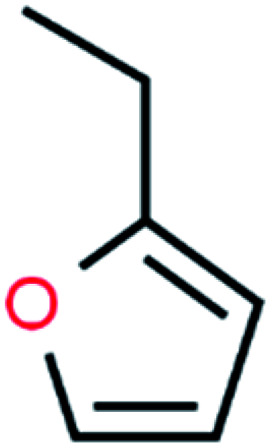
3	8.699	3.96	C_6_H_12_	Tetramethyl ethylene	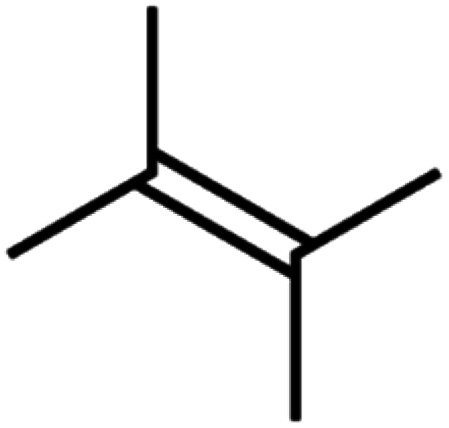
4	9.187	1.26	C_7_H_12_O	Acetyl cyclopentane	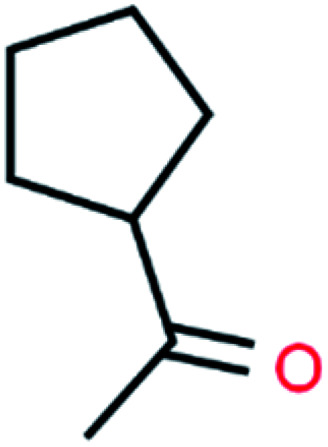
5	10.47	2.46	C_7_H_14_	2,4-Dimethyl-1-pentene	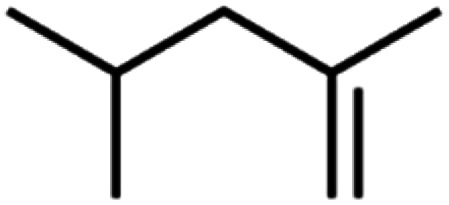
6	10.787	1.14	C_8_H_14_O	2-Methyl-2-hepten-4-one	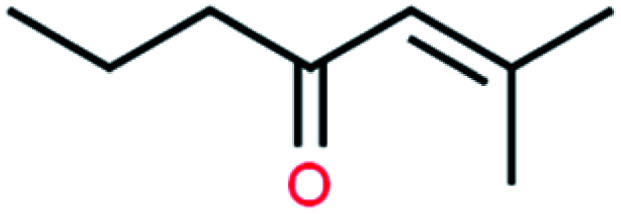
7	11.631	6.67	C_8_H_18_	4-Methylheptane	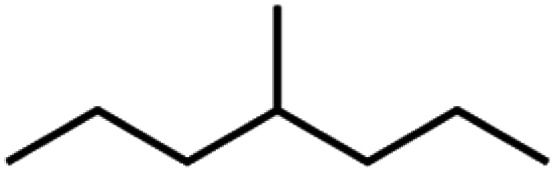
8	11.844	1.03	C_9_H_16_	1,2,4,4-Tetramethylcyclopentene	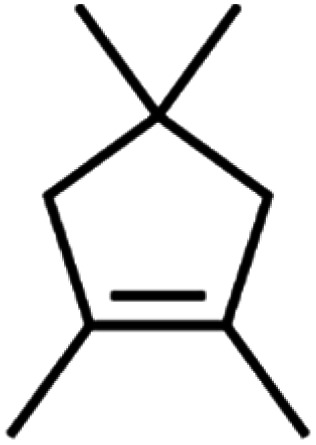
9	12.013	0.58	C_9_H_18_	(1*r*)-1,3,5-trimethylcyclohexane	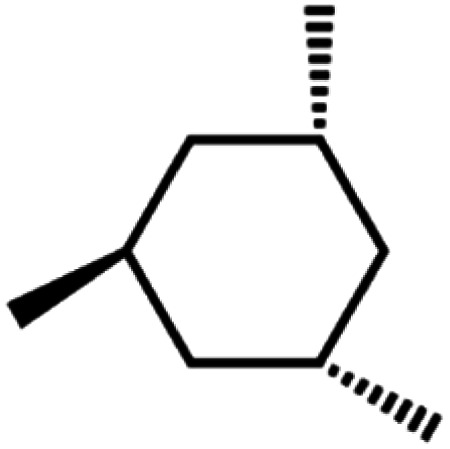
10	12.379	1.08	C_10_H_20_	1-Decene	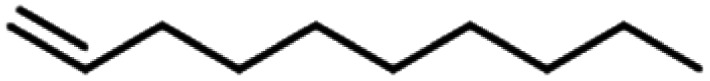
11	14.052	1.67	C_10_H_20_	2-Methyl-2-nonene	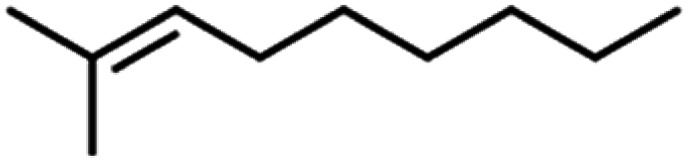
12	14.317	4.16	C_10_H_20_	1-Decene	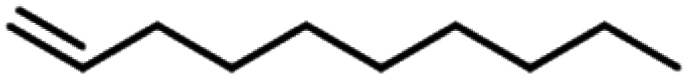
13	15.237	8.31	C_10_H_20_	2-Methyl-2-nonene	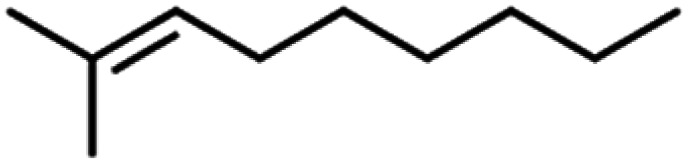
14	15.841	0.79	C_10_H_22_	Decane	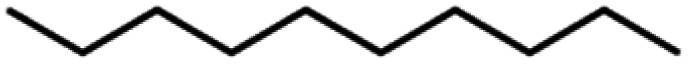
15	16.189	2.31	C_11_H_22_	*n*-1-Undecene	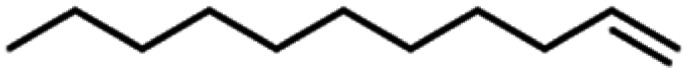
16	16.579	1.99	C_11_H_24_	*n*-Undecane	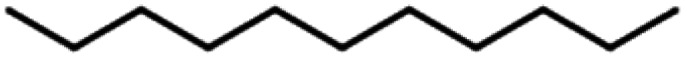
17	16.848	1.16	C_12_H_24_	1-Dodecene	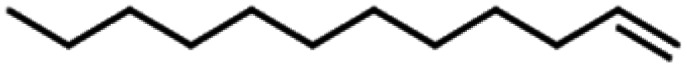
18	17.002	15.01	C_12_H_26_	*n*-Dodecane	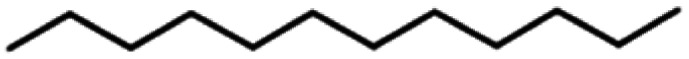
19	17.72	1.13	C_13_H_26_	1-Tridecene	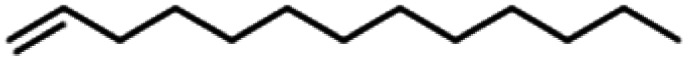
20	18.188	1.14	C_13_H_28_	Tridecane	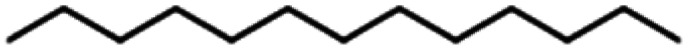
21	18.449	10.78	C_14_H_28_	1-Tetradecene	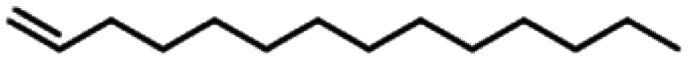
22	18.727	6.52	C_14_H_30_	Tetradecane	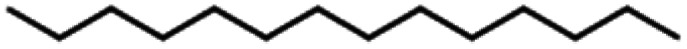
23	19.63	6.78	C_15_H_30_	1-Pentadecene	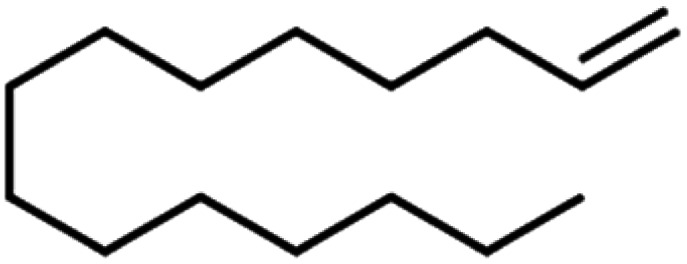
24	20.456	1.86	C_15_H_32_	Pentadecane	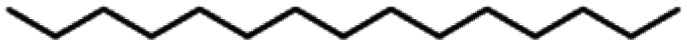
25	21.822	0.98	C_16_H_32_	1-Hexadecene	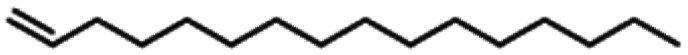
26	22.162	1.06	C_16_H_34_	*n*-Hexadecane	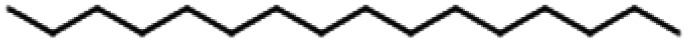
27	22.359	2.41	C_18_H_36_	1-Octadecene	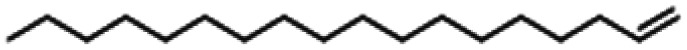
28	24.431	1.46	C_18_H_36_	1-Octadecene	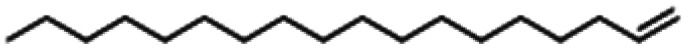
29	24.551	1.61	C_17_H_36_	*n*-Heptadecane	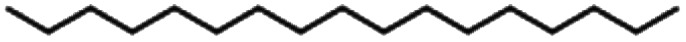
30	30.063	1.85	C_19_H_40_	Nonadecane	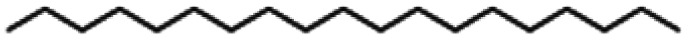
31	30.231	0.97	C_20_H_42_	Eicosane	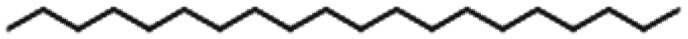
32	30.409	1.84	C_20_H_42_	Eicosane	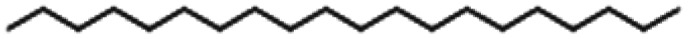
33	30.951	0.82	C_21_H_44_	*n*-Heneicosane	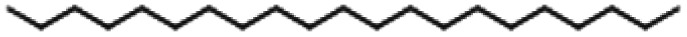
34	31.374	0.54	C_22_H_46_	Docosane	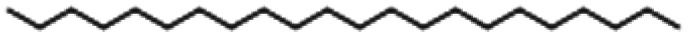
35	32.712	0.67	C_24_H_50_	*n*-Tetracosane	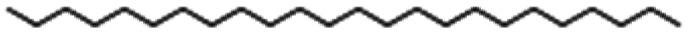
36	33.914	0.34	C_25_H_52_	Pentacosane	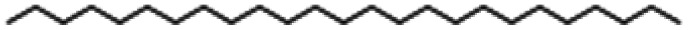

## 4. Experimental setup

**Fig. 6:**
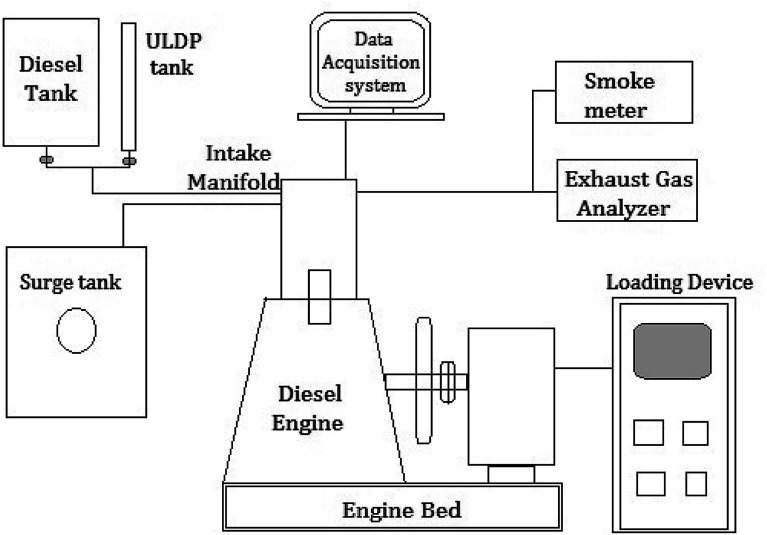
Schematic experimental layout of DI diesel engine setup.

The blends of ULDP oil such as ULDP20 (20% of ULDP + 80% of diesel), ULDP40 (40% of ULDP + 60% of diesel), ULDP60 (60% of ULDP + 40% of diesel), and ULDP80 (80% of ULDP + 20% of diesel) were prepared with diesel on a volume basis. The engine trials were conducted on a four-stroke single-cylinder DI diesel engine fueled with diesel, neat ULDP oil and its blends. The schematic layout of the DI diesel engine setup is shown in Fig. 6 and its detailed specifications are listed in Table 5. An experimental test was carried out in a single-cylinder four-stroke compression engine with a rated speed of 1500 rpm. It was coupled with an electrical dynamometer for loading. Several operating conditions of the engine are unchanged, with an intake air temperature of 27 °C and injection pressure 200 bar.^32^ However, thermocouples were placed at different positions to measure the intake air temperature, exhaust gas temperature, and fuel inlet temperature. The smoke density was measured using an AVL smoke meter, and the exhaust gas analyzer measured the amount of CO, CO_2_, NOx, and HC in ppm.^33^ An experimental investigation was carried out in an air-cooled single-cylinder four-stroke diesel engine with a data acquisition system at different loading conditions. Performance and emission characteristics were observed on varying loads from 0 to 100% for ULDP20, ULDP40, ULDP60, ULDP80, and neat ULDP oil. The trials were repeated twice to obtain optimum readings.

**Table 5:** Engine specifications

Make & model	Kirloskar, TAF1
Engine type	CI, four stroke, direct injection, vertical air-cooled diesel engine
Bore (mm)	87.5
Stroke (mm)	110
Compression ratio	17.5
Rated power @ 1500 rpm (kW)	4.4
Nozzle operating pressure (bar)	200
Injection timing (CA)	23 °bTDC

## 5. Result and discussion

### 5.1 Combustion analysis

#### 5.1.1 Cylinder pressure

**Fig. 7:**
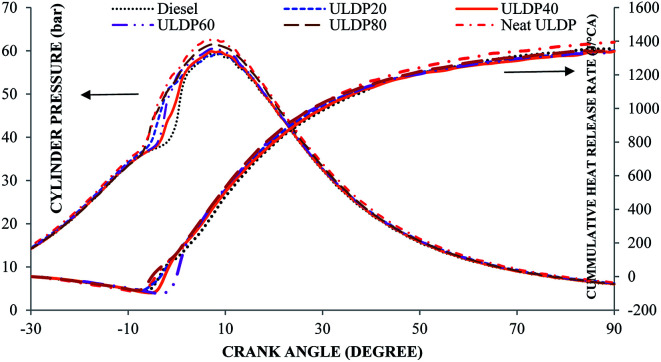
Variation of cylinder pressure (bar) and cumulative heat release rate (J/°CA) with crank angle (degree) at full load.

The variation of cylinder pressure with the crank angle for various test fuels like diesel, neat ULDP oil, and its blends at full load is depicted in Fig. 7. The quantity of fuel injected in the uncontrolled combustion region and delay period influences the cylinder peak pressure. At the maximum load condition, the cylinder peak pressures are 58.97 bar for diesel, 62.60 bar for neat ULDP oil, 59.31 bar for ULDP20, 59.85 bar for ULDP40, 60.44 bar for ULDP60, and 61.43 bar for ULDP80 blends. The cylinder pressure of neat ULDP oil is 6.15% higher than diesel due to the higher heat release rate and longer ignition delay. It was observed that the cylinder peak pressure increases gradually with the increase in plastic oil blends, which is because the increasing number of aromatic bonds in the higher blends of plastic oil require more energy to break down the structure.^34^ However, the cylinder peak pressure of ULDP20 is 5.24% lower than that of neat ULDP oil and much closer (0.58%) to that of diesel fuel at full load.

#### 5.1.2 Cumulative heat release rate

Variation in the cumulative heat release rate with the crank angle for different test fuels like diesel, neat ULDP oil, and its blends at maximum load is depicted in Fig. 7. The total heat released during the combustion process provides information on the cumulative heat release, which gives insight into the progress of combustion. At a full load condition, the cumulative heat release rates are 1359.5 J/°CA for diesel, 1394.4 J/°CA for neat ULDP oil, 1344.5 J/°CA for ULDP20, 1340.1 J/°CA for ULDP40, 1342.0 J/°CA for ULDP60, and 1349.0 J/°CA for ULDP80 blends. At a maximum load, the cumulative heat release rate of neat (100%) ULDP is 2.56% higher than diesel fuel due to a higher energy content and rise in the in-cylinder peak pressure. However, the cumulative heat release rate of ULDP 20 is 3.57% lower than neat ULDP oil and much closer (1.11%) to that of diesel fuel at a full load.

#### 5.1.3 Heat release rate

**Fig. 8:**
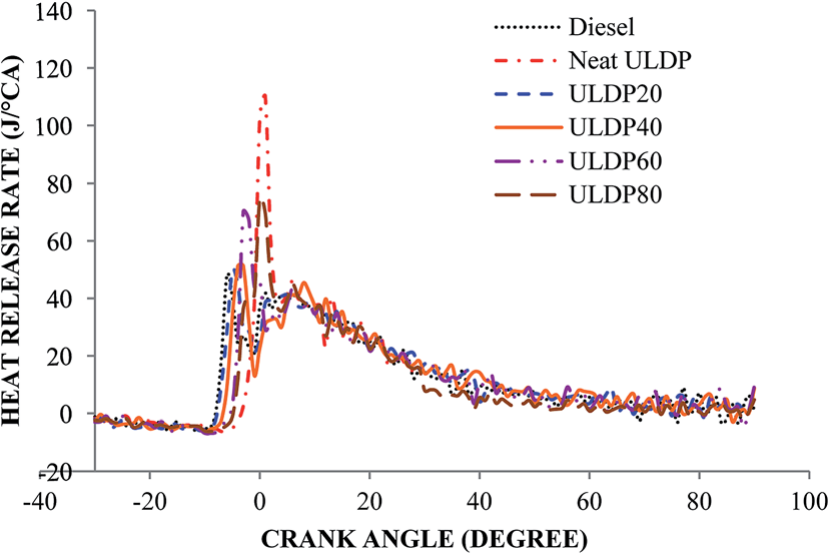
Variation of heat release rate (J/°CA) with crank angle (degree) at full load.

Variation in the heat release rate with the crank angle for selected test fuels like diesel, neat ULDP oil, and its blends at maximum load is depicted in Fig. 8. Heat release gives a clear picture of the performance, nitrogen oxides formation, and engine cooling requirements. At full load condition, the maximum heat release rates are 47.82 J/°CA for diesel, 102.9 J/°CA for neat ULDP oil, 49.86 J/°CA for ULDP20, 51.26 J/°CA for ULDP40, 67.37 J/°CA for ULDP60, and 74.49 J/°CA for ULDP80 blends. At the full load maximum, the heat release rate of neat ULDP oil is 115.15% higher than that of diesel due to a longer ignition delay that causes a greater premixed combustion at higher percentage blends.^35^ However, premixed combustion in the rapid burning phase leads to higher heat release and cylinder pressure. Further, the maximum heat release rate of ULDP20 is 51.54% lower than neat (100%) ULDP and 4.2% higher than that of diesel fuel at full load.

#### 5.1.4 Ignition delay

**Fig. 9:**
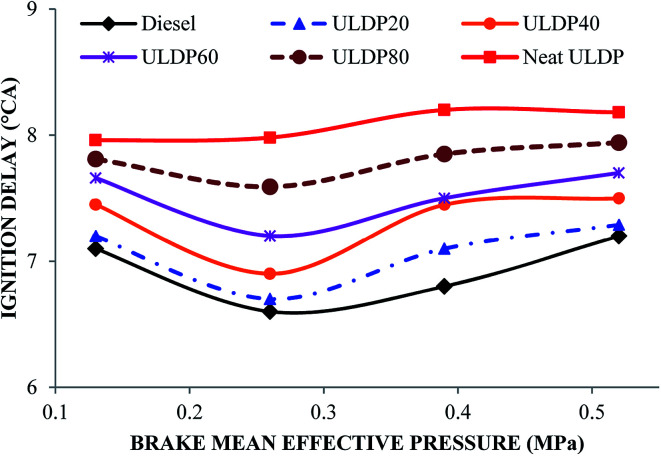
Variation of ignition delay (°CA) with brake mean effective pressure (MPa).

The differences in ignition delay with the brake mean effective pressure for selected test fuels like diesel, neat (100%) ULDP oil and its blends at all loads are depicted in Fig. 9. The ignition delay is defined as the delay period between the start of fuel injection and the start of combustion, and it can be read from the heat release rate curves.^36^ The ignition delay for diesel fuel increases from 7.1 °CA at (25% load) low load to 7.2 °CA at (100%) full load. In the case of ULDP20, it varies from 7.2 °CA at a low load to 7.29 °CA at full load. In the case of neat ULDP oil, it varies from 7.96 °CA at a low load to 8.18 °CA at full load. At full load condition, the ignition delay is 7.2 °CA for diesel, 8.18 °CA for neat ULDP oil, 7.29 °CA for ULDP20, 7.50 °CA for ULDP40, 7.70 °CA for ULDP60, and 7.94 °CA for ULDP80 blends. The longer ignition delay due to a higher heat release rate results in a higher cylinder pressure. The lower viscosity of neat ULDP oil affects the spray characteristics during injection that influences the longer ignition delay due to poor ignition.^37^ The ignition delay period calculated for ULDP20 at 25%, 50%, 75% and 100% loads were 7.2 °CA, 6.7 °CA, 7.1 °CA and 7.29 °CA.

#### 5.1.5 Combustion duration

**Fig. 10:**
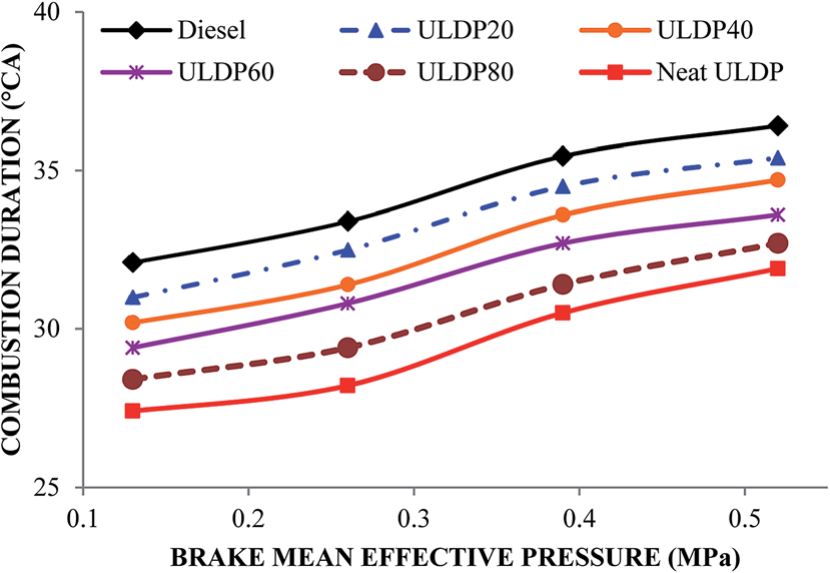
Variation of combustion duration (°CA) with brake mean effective pressure (MPa).

The differences in combustion duration with the brake mean effective pressure for selected test fuels like diesel, neat (100%) ULDP oil and its blends at all loads are depicted in Fig. 10. The combustion duration (CD) is a measure of time between the start of combustion (SOC) and end of combustion (EOC) in the p-theta diagram. The CD for diesel fuel increased from 32.1 °CA at (25% load) low load to 36.4 °CA at (100% load) full load. In the case of ULDP20, it varies from 31 °CA at a low load to 35.4 °CA at full load. In the case of neat ULDP oil, it varies from 27.4 °CA at a low load to 31.9 °CA at full load. At full load condition, the CD is 36.4 °CA for diesel, 31.9 °CA for neat ULDP oil, 35.4 °CA for ULDP20, 34.7 °CA for ULDP40, 33.6 °CA for ULDP60, and 32.7 °CA for ULDP80 blends. The CD decreased with an increase in ULDP oil concentration in the blends owing to a higher heat release rate and longer ignition delay. In a controlled burning phase, the higher blends of ULDP oil exhibit more rapid combustion than diesel fuel by producing an enormous amount of heat at the initial stage of SOC, thus making the duration shorter.^38^. The CD period calculated for ULDP20 at 25%, 50%, 75% and 100% loads were 31 °CA, 32.5 °CA, 34.5 °CA and 35.2 °CA.

#### 5.1.6 Cylinder peak pressure

**Fig. 11:**
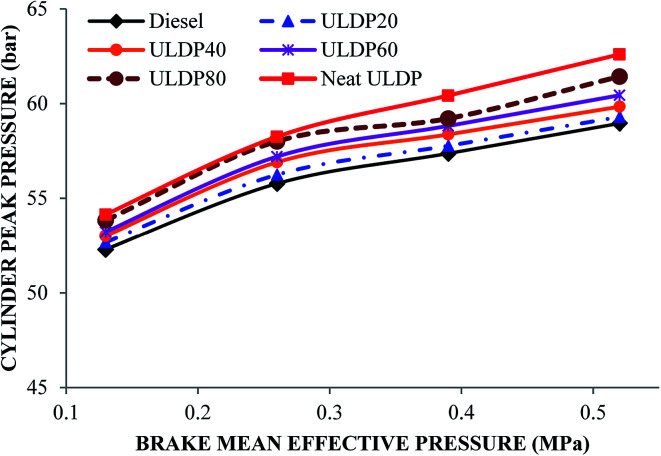
Variation of cylinder peak pressure (bar) with brake mean effective pressure (MPa).

The variation of cylinder peak pressure with brake mean effective pressure for various test fuels like diesel, neat ULDP oil, and its blends at all loads are shown in Fig. 11. At full load condition, the cylinder peak pressures are 58.97 bar for diesel, 62.60 bar for neat ULDP oil, 59.31 bar for ULDP20, 59.85 bar for ULDP40, 60.44 bar for ULDP60, and 61.43 bar for ULDP80 blends. For neat (100%) ULDP, the cylinder peak pressure is 6.15% higher than that of diesel due to its slow chemical reaction. In the earlier stage of combustion, the amount of fuel decides the cylinder peak pressure reliant on the delay period.^37^ However, the cylinder peak pressure of ULDP 20 is 5.25% lower than neat ULDP oil and much closer (0.57%) to that of diesel fuel at full load. The cylinder peak pressures for ULDP20 at 25%, 50%, 75%, and 100% loads were 52.68 bar, 56.26 bar, 57.78 bar, and 59.31 bar, respectively.

### 5.2 Performance characteristics

#### 5.2.1 Brake thermal efficiency

**Fig. 12:**
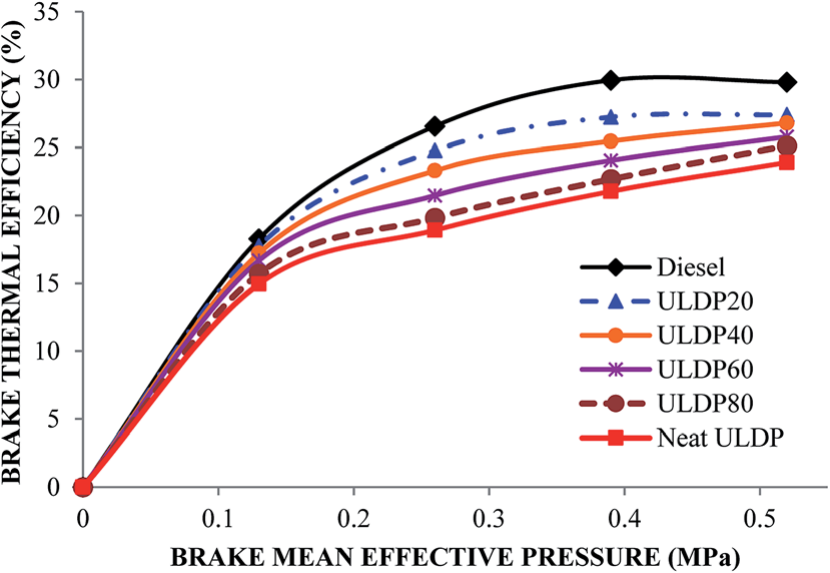
Brake thermal efficiency variations (%) with brake mean effective pressure (MPa).

The differences in the brake thermal efficiency with brake mean effective pressure for diesel, neat ULDP oil, and its blends at all loads are depicted in Fig. 12. The brake thermal efficiencies at full load are 29.8% for diesel, 23.9% for neat ULDP oil, 27.3% for ULDP20, 26.7% for ULDP40, 25.7% for ULDP60, and 25.1% for ULDP80 blends. For all experimental fuels, the variation in brake thermal efficiency increases with an increase in load. The BTE of neat ULDP oil is 19.78% lower than that of standard diesel fuel due to the slow chemical reaction because of the unsaturated double bond present in the molecular structure of the aromatic compounds.^39^ However, the brake thermal efficiency of ULDP20 is 14.59% higher than neat ULDP oil and marginally less than diesel at full load. The proportional increase in carbon atoms due to the blending of fuels increases the boiling point, which leads to an increase in engine efficiency. The brake thermal efficiencies observed for ULDP20 at 25%, 50%, 75% and 100% loads are 17.75%, 24.78%, 27.23% and 27.39%, respectively. The ULDP20 blend performed very closely to that of standard diesel fuel.

#### 5.2.2 Brake specific fuel consumption

**Fig. 13:**
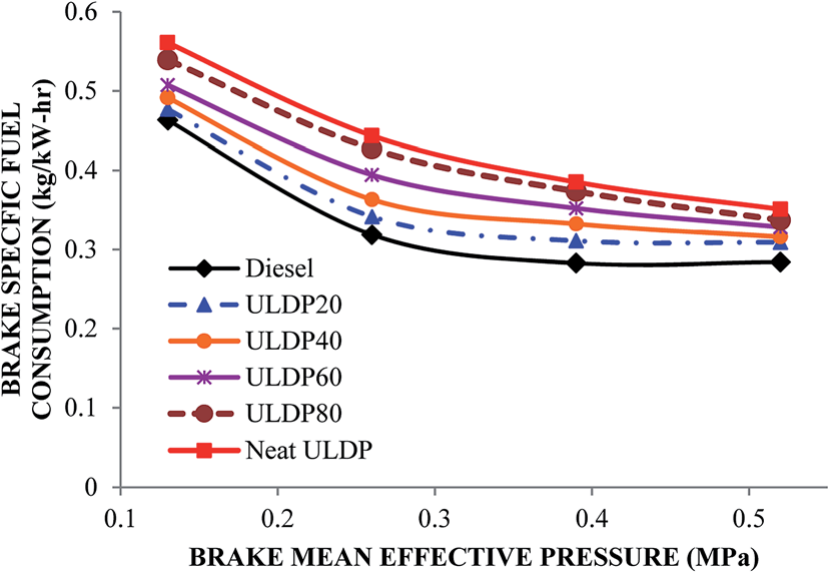
Variation of brake specific fuel consumption (kg kW^−1^ h^−1^) with brake mean effective pressure (MPa).

The differences in brake specific fuel consumption with brake mean effective pressure for various test fuels like diesel, neat ULDP oil, and its blends at all loads are depicted in Fig. 13. The BSFC decreases with an increase in load. At the maximum load condition, the brake specific fuel consumptions are 0.284 kg kW^−1^ h^−1^ for diesel, 0.351 kg kW^−1^ h^−1^ for neat ULDP oil, 0.309 kg kW^−1^ h^−1^ for ULDP20, 0.316 kg kW^−1^ h^−1^ for ULDP40, 0.328 kg kW^−1^ h^−1^ for ULDP60, and 0.337 kg kW^−1^ h^−1^ for ULDP80 blends. The BSFC of neat ULDP oil is 23.5% higher than that of standard diesel due to its low viscosity and density when compared to other blends. This indicates that neat ULDP oil is more volatile than other tested fuels.^40^ The high volatility of neat ULDP oil reduces the volumetric efficiency and causes vapor lock under hot climatic conditions. However, the brake specific fuel consumption of ULDP20 is 11.91% lower than neat ULDP oil and 8.79% higher than standard diesel fuel at full load. The BSFC observed for ULDP20 at 25%, 50%, 75% and 100% loads were 0.476 kg kW^−1^ h^−1^, 0.341 kg kW^−1^ h^−1^, 0.311 kg kW^−1^ h^−1^ and 0.309 kg kW^−1^ h^−1^, respectively.

#### 5.2.3 Exhaust gas temperature

**Fig. 14:**
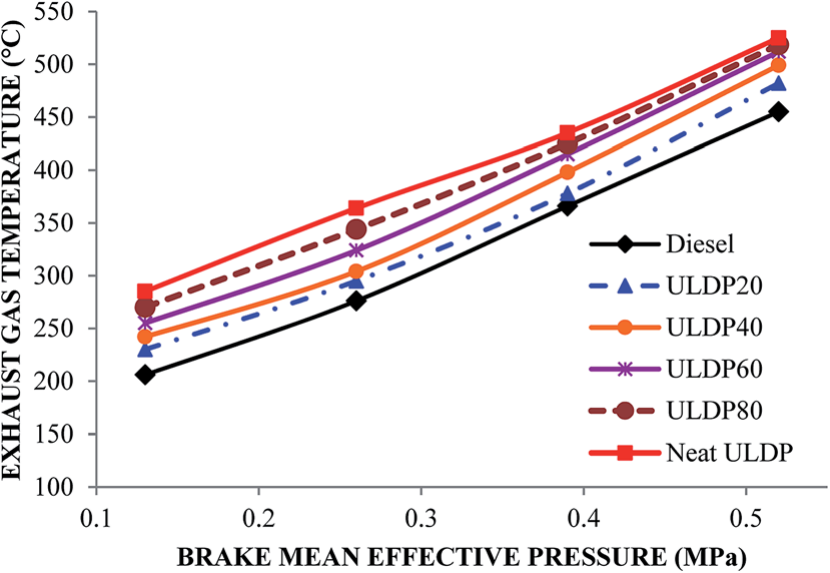
Variation of exhaust gas temperature (°C) with brake mean effective pressure (MPa).

The differences in exhaust gas temperature with brake mean effective pressure for selected test fuels like diesel, neat ULDP oil, and its blends at all loads are depicted in Fig. 14. The increase in the amount of fuel injected into the combustion chamber rises with an increasing load and resulted in a higher exhaust gas temperature. At full load condition, the exhaust gas temperature is 455 °C for diesel, 525 °C for neat ULDP oil, 482 °C for ULDP20, 499 °C for ULDP40, 512 °C for ULDP60, and 518.5 °C for ULDP80 blends. The exhaust gas temperature of neat ULDP oil is 15.38% higher than that of diesel due to the abnormal heat release rate. Better atomization of fuel induces the premixed combustion, which leads to higher exhaust gas temperatures. However, the EGT of ULDP20 is 13.33% lower than neat ULDP oil and 5.93% higher than that of standard diesel fuel at maximum load. The exhaust gas temperatures observed for ULDP20 at 25%, 50%, 75% and 100% loads were 230 °C, 295 °C, 378 °C, and 482 °C, respectively.

### 5.3 Emission characteristics

#### 5.3.1 Nitrogen oxides

**Fig. 15:**
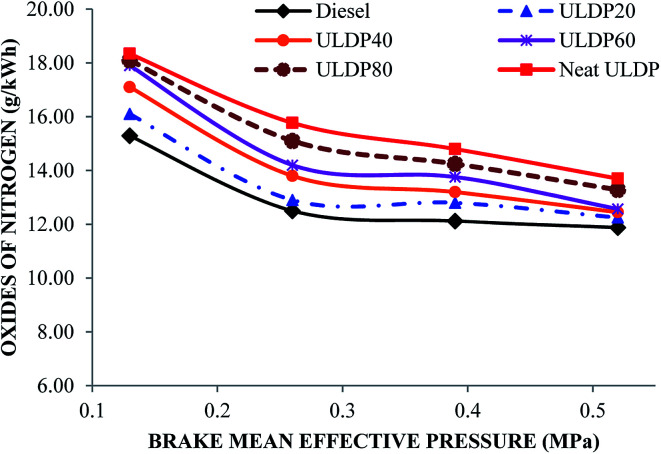
Differences in nitrogen oxides (g kW^−1^ h^−1^) with brake mean effective pressure (MPa).

The various nitrogen oxides with brake mean effective pressure for various test fuels like diesel, neat ULDP oil, and its blends at all loads are depicted in Fig. 15. At full load condition, the nitrogen oxide emissions are 11.88 g kW^−1^ h^−1^ for diesel, 13.70 g kW^−1^ h^−1^ for neat ULDP oil, 12.25 g kW^−1^ h^−1^ for ULDP20, 12.45 g kW^−1^ h^−1^ for ULDP40, 12.57 g kW^−1^ h^−1^ for ULDP60, and 13.28 g kW^−1^ h^−1^ for ULDP80 blends. The proportional increase in the concentration of ULDP leads to an increase in the nitrogen oxides emission. The nitrogen oxide emission of neat ULDP oil is 15.31% higher than that of diesel due to the higher aromatic content with a peculiar ring structure, which results in a higher heat release rate. This occurs due to the increase in in-cylinder temperature and high peak pressure during combustion. However, the NOx from ULDP20 is 10.58% lower than neat ULDP oil and marginally (3.11%) higher than that of standard diesel fuel at maximum load. Premixed combustion creates a longer ignition delay, which enhances the NOx emission.^41^ The nitrogen oxides observed for ULDP20 at 25%, 50%, 75%, and 100% loads were 16.10 g kW^−1^ h^−1^, 12.90 g kW^−1^ h^−1^, 12.80 g kW^−1^ h^−1^ and 12.25 g kW^−1^ h^−1^, respectively.

#### 5.3.2 Unburned hydrocarbon

**Fig. 16:**
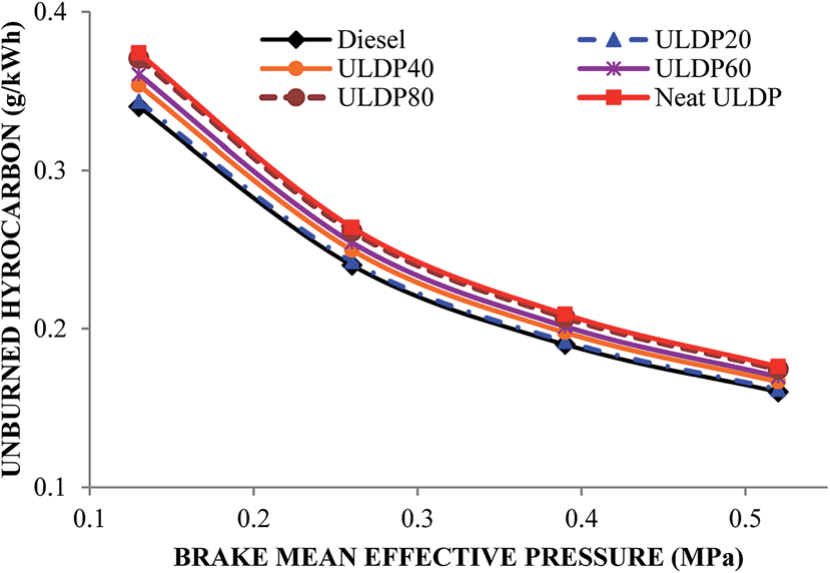
Variation of unburned hydrocarbon (g kW^−1^ h^−1^) with brake mean effective pressure (MPa).

The unburned hydrocarbon variations with brake mean effective pressure for selected fuels like diesel, neat (100%) ULDP oil and its blends at all loads are depicted in Fig. 16. At full load condition, the unburned hydrocarbon emissions are 0.160 g kW^−1^ h^−1^ for diesel, 0.1761 g kW^−1^ h^−1^ for neat ULDP oil, 0.161 g kW^−1^ h^−1^ for ULDP20, 0.166 g kW^−1^ h^−1^ for ULDP40, 0.169 g kW^−1^ h^−1^ for ULDP60, and 0.174 g kW^−1^ h^−1^ for ULDP80 blends. The unburned hydrocarbon emission of neat ULDP oil is 10% higher than that of diesel due to a lack of oxygen content in the rich mixture. It was found that at higher loads, the unburned hydrocarbon emission was lower when compared to lower loads due to the increase in combustion temperature. For higher blends, an increased unburned hydrocarbon is observed due to the unsaturated hydrocarbons. However, the unburned hydrocarbon emission of ULDP20 is 8.18% less than that of neat ULDP oil and marginally (1%) higher than diesel at maximum load. The UHC examined for ULDP20 at 25%, 50%, 75% and 100% loads were 0.343 g kW^−1^ h^−1^, 0.242 g kW^−1^ h^−1^, 0.191 g kW^−1^ h^−1^ and 0.161 g kW^−1^ h^−1^, respectively.

#### 5.3.3 Carbon monoxide

**Fig. 17:**
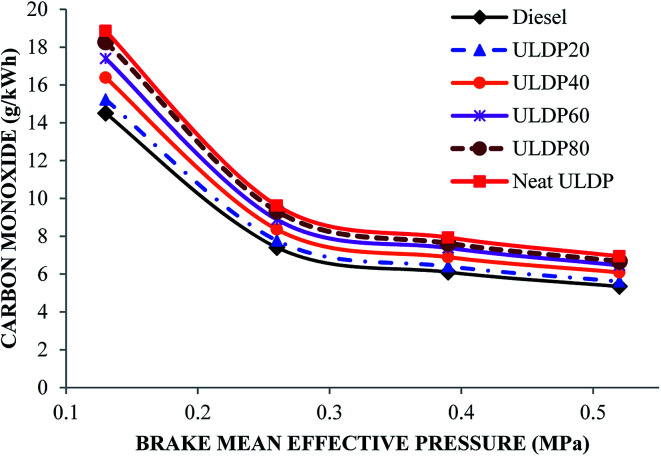
Variation of carbon monoxide (g kW^−1^ h^−1^) with brake mean effective pressure (MPa).

Differences in the carbon monoxide with brake mean effective pressure for various test fuels like diesel, neat ULDP oil, and its blends at all loads are depicted in Fig. 17. At full load condition, the carbon monoxide emissions are 5.340 g kW^−1^ h^−1^ for diesel, 6.942 g kW^−1^ h^−1^ for neat ULDP oil, 5.607 g kW^−1^ h^−1^ for ULDP20, 6.087 g kW^−1^ h^−1^ for ULDP40, 6.461 g kW^−1^ h^−1^ for ULDP60, and 6.675 g kW^−1^ h^−1^ for ULDP80 blends. CO emissions gradually decrease with an increase in loads due to better combustion caused by an increase in the in-cylinder temperature and pressure. The CO emissions of neat ULDP oil are 30% higher than standard diesel due to the presence of more aromatic compounds and insufficient oxygen content, which causes longer ignition delays. However, the CO emission of ULDP20 is 23.80% less than neat ULDP oil and 5% higher than that of diesel fuel at full load. The carbon monoxide emissions observed for ULDP20 at 25%, 50%, 75% and 100% loads were 15.225 g kW^−1^ h^−1^, 7.770 g kW^−1^ h^−1^, 6.405 g kW^−1^ h^−1^ and 5.607 g kW^−1^ h^−1^, respectively.

#### 5.3.4 Carbon dioxide

**Fig. 18:**
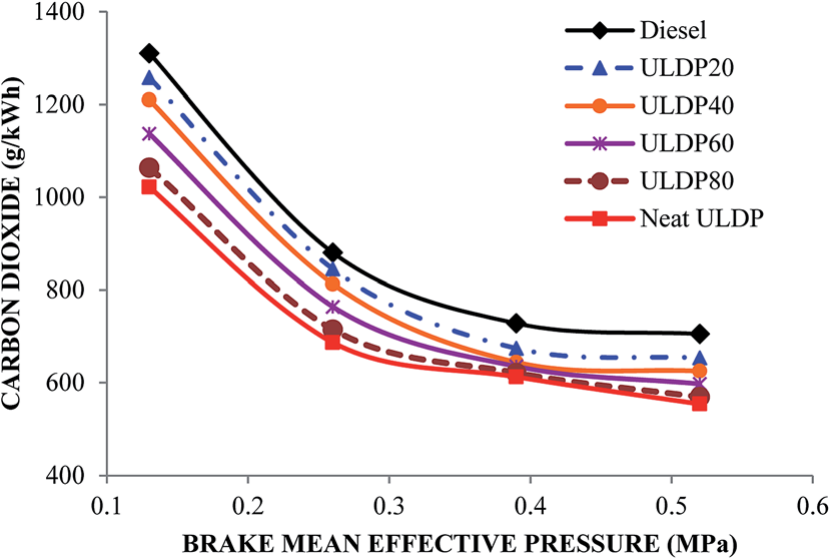
Carbon dioxide variations (g kW^−1^ h^−1^) with brake mean effective pressure (MPa).

The carbon dioxide (CO_2_) variations with brake mean effective pressure for selected test fuels like diesel, neat (100%) ULDP oil and its blends at all loads are depicted in Fig. 18. At full load condition, the carbon dioxide emissions are 705 g kW^−1^ h^−1^ for diesel, 553.8 g kW^−1^ h^−1^ for neat ULDP oil, 653.2 g kW^−1^ h^−1^ for ULDP20, 624.8 g kW^−1^ h^−1^ for ULDP40, 597.11 g kW^−1^ h^−1^ for ULDP60, and 568.71 g kW^−1^ h^−1^ for ULDP80 blends. The carbon dioxide emissions of neat ULDP oil are 21.44% less than standard diesel due to the lack of oxygen, thus leading to partial oxidation. However, the carbon dioxide emission of ULDP20 is 17.94% higher than neat ULDP oil and 7.34% lesser than that of diesel fuel at full load. The carbon dioxide emissions observed for ULDP20 at 25%, 50%, 75% and 100% loads were 1257.6 g kW^−1^ h^−1^, 844.8 g kW^−1^ h^−1^, 674 g kW^−1^ h^−1^ and 653.2 g kW^−1^ h^−1^, respectively.

#### 5.3.5 Smoke

**Fig. 19:**
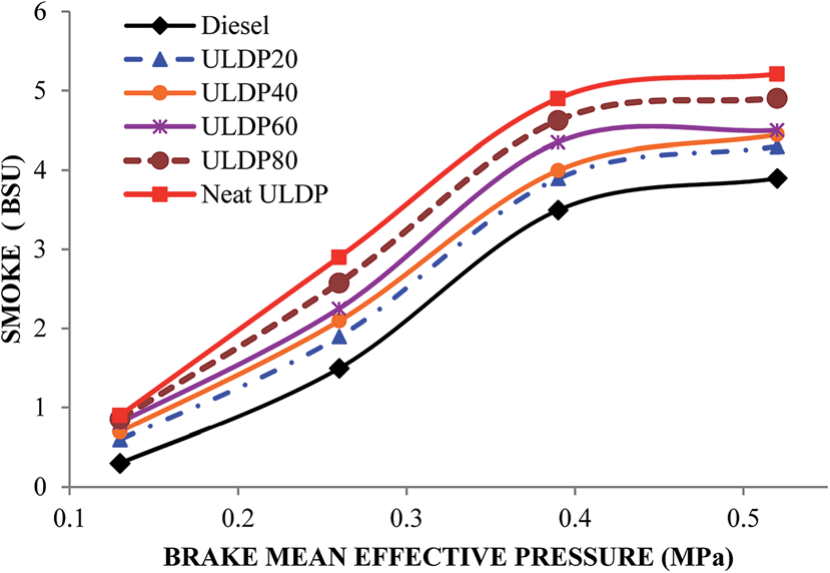
Variation of smoke (BSU) with brake mean effective pressure (MPa).

The differences in smoke with BMEP for selected test fuel samples like diesel, neat ULDP oil, and its blends at all loads are depicted in Fig. 19. At full load condition, the smoke emissions are 3.90 BSU for diesel, 5.21 BSU for neat ULDP oil, 4.30 BSU for ULDP20, 4.45 BSU for ULDP40, 4.50 BSU for ULDP60, and 4.90 BSU for ULDP80 blends. The smoke emission of neat ULDP oil is 33.58% higher than that of diesel due to the rich quality of fuel present during the combustion phase, which leads to a longer ignition delay.^38^ However, the smoke emission of ULDP20 is 17.46% less than neat ULDP oil and 10.19% higher than diesel fuel at maximum load. The smoke emission observed for ULDP20 at 25%, 50%, 75%, and 100% loads were 0.60 BSU, 1.90 BSU, 3.90 BSU, and 4.30 BSU, respectively.

### 6.0 Uncertainty and error analysis

Uncertainty analysis is critical to prove the accuracy of the experiment. During experimental analysis, ambiguities and uncertainties were found. However, this may occur due to calibration error, human error, environmental conditions, and instrument components.^42^ The estimated uncertainty values, range, and accuracy of the instruments are shown in Table 6. The root sum square method was used to estimate the accurate uncertainty limits.
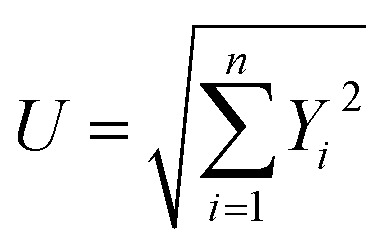
where *U* is the total percentage uncertainty, *Y*_*i*_ are the individual uncertainties of parameters, *Y*_1_ is the brake power uncertainty percentage, *Y*_2_ is the total fuel consumption uncertainty percentage, *Y*_3_ is the exhaust gas temperature uncertainty percentage, *Y*_4_ is the nitrogen oxides emission uncertainty percentage, *Y*_5_ is the carbon monoxide emission uncertainty percentage, *Y*_6_ is the unburned hydrocarbon emission uncertainty percentage, and *Y*_7_ is the smoke uncertainty percentage.
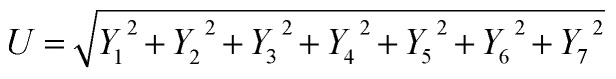

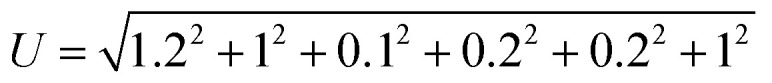
*U* = ±1.89%

**Table 6:** List of instruments with percentage uncertainties, accuracy, and range

Instruments	Range	Accuracy	Percentage uncertainties
Burette for fuel measurement	—	+0.1 cc to −0.1 cc	+1 to −1
Digital stop watch	—	+0.5 s to −0.5 s	+0.2 to −0.2
Manometer	—	+1 mm to −1mm	+1 to −1
Crank angle encoder	0–100 bar	+1° to −1°	+0.1 to −0.1
Load indicator	0–100 kg	+0.1 kg to −0.1 kg	+0.15 to −0.15
Speed measuring unit	0–1000 rpm	+10 rpm to −10 rpm	+0.15 to −0.15
Exhaust gas temperature indicator	0–1000 °C	+1 °C to −1 °C	+0.1 °C to −0.1 °C
Smoke level measuring instrument – AVL 415	BSU 0–10	+0.15 to −0.15	+1 to −1
Gas analyser – AVL DI GAS 444	CO 0–10%	+0.02% to −0.02%	+0.2 to −0.2
	CO_2_ 0–20%	+0.03% to −0.03%	+0.15 to −0.15
	HC 0–20 000 ppm	+10 ppm to −10 ppm	+0.2 to −0.2
	NOx 0–5000 ppm	+10 ppm to −10 ppm	+0.2 to −0.2

## 6. Conclusion

Without any diesel engine modifications, the following conclusions were derived from the experimental work conducted with standard diesel, neat (100%) plastic oil and its blends.

• The compression ignition (CI) engine was run on neat plastic oil without any engine modification. The longer ignition delay and very high premixed combustion restricted the usage of neat ULDP oil in diesel engines for prolonged run usage. The unusual trend of uncontrolled combustion, longer combustion duration, and poor performance limited further analysis.

• ULDP20 performed very closely to that of diesel in the case of brake thermal efficiency, and it varied from 17.75% to 27.39%.

• The marginal increase in heat release rate was observed for ULDP20 at the normal operating condition. However, the ignition delay period was very close to that of diesel in the case of ULDP20, and it varied from 7.2 °CA to 7.29 °CA.

• In the case of ULDP 20, unburned hydrocarbon, NOx and CO emissions were marginally higher by 1%, 3.11%, and 5%, respectively, at full load compared with diesel fuel.

• The smoke emission of ULDP20 was very similar to standard diesel fuel operation. For ULDP20, the smoke emissions varied from 0.60 BSU to 4.30 BSU.

Based on the above conclusions, ULDP20 can be used as fuel for DI diesel engines without any engine modifications.

## Conflicts of interest

There are no conflicts to declare.

## Supplementary Material
